# HPV16 E2 protein possesses intrinsic helicase activity and sterically hinders E1 function through direct interaction

**DOI:** 10.1016/j.jbc.2026.113143

**Published:** 2026-05-20

**Authors:** Ping Xu, Shuning Cai, Lihong Zhang, Yueqi Wu, Ke Xu, Yigang Tong, Shan Xu

**Affiliations:** 1 BAICSM, State Key Laboratory of Green Biomanufacturing, College of Life Science and Technology, Beijing University of Chemical Technology, Beijing, China; 2 Department of Pharmacy, The Second Qilu Hospital of Shandong University, Jinan, China

**Keywords:** HPV16, E2, E1, helicase, ATP

## Abstract

HPV16 E2 protein is a key regulatory protein essential for viral replication, yet no enzymatic activity has been attributed to it until now. In this study, we report for the first time that E2 possesses intrinsic ATP-dependent DNA unwinding activity. Mutational analysis identified residues K299, Y303, and K306 as critical for this helicase function. We further demonstrate that podophyllotoxin directly binds to E2 and inhibits its unwinding activity with an IC_50_ of 0.11±0.03 μM, mediated primarily by residues H320 and Q342. Comparative analysis revealed that the ATPase and helicase activities of E2 are considerably weaker than those of E1. Notably, we discovered that E2 potently inhibits the helicase activity of E1. This suppression is facilitated by the N-terminal domain of E2 (amino acids 1–245) through direct interaction with E1, with residue E39 playing a critical role. Our findings not only unveil a previously unrecognized enzymatic function of E2 but also suggest its role as a potential antiviral target. Moreover, the observed inhibitory effect of E2 on E1 highlights a novel regulatory mechanism for HPV DNA replication.

**Figure:**
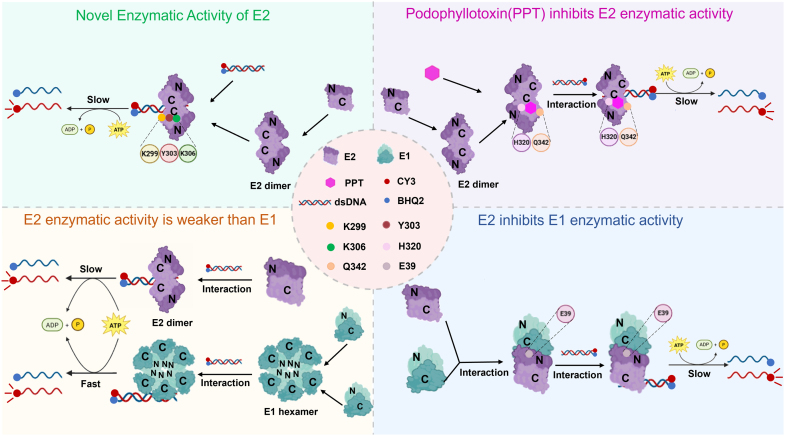
E2 protein has traditionally been recognized primarily for its DNA-binding and transcriptional regulatory functions. This study provides the first evidence that E2 protein possesses intrinsic enzymatic activity, identifies a small-molecule inhibitor targeting this activity, and reveals a novel mechanism of E1-E2 interaction.•HPV16 E2 protein exhibits ATPase and helicase activities•Identification of key amino acid residues for HPV16 E2 protein enzymatic activity•PPT effectively inhibits E2 protein helicase activity in vitro•E2 protein exhibits weaker enzymatic activity than E1 and inhibits E1 helicase activity•E2 inhibits E1 helicase activity through protein-protein interaction

Human papillomavirus (HPV) is one of the most common sexually transmitted infections worldwide. Recent epidemiological data from the World Health Organization (WHO) indicate that approximately 11 to 15% of reproductive-aged women (15–49 years) harbor genital HPV infections, of which 4 to 7% are attributed to high-risk genotypes, constituting a substantial public health burden (1). HPV-associated malignancies account for an estimated 342,000 annual deaths globally, with cervical cancer representing 88% of this burden, disproportionately affecting low- and middle-income countries (2, 3, 4, 5). These data highlight the significant impact of HPV infection on health equity, particularly in regions with limited healthcare resources. HPV is a non-enveloped double-stranded DNA virus that specifically infects cutaneous and mucosal epithelial tissues (6, 7, 8). More than 130 genotypes have been characterized, each exhibiting distinct pathological manifestations ranging from benign lesions (*e.g.*, common warts and condylomata acuminata) to malignant transformations (9, 10, 11). High-risk genotypes, notably HPV16 and HPV18, are established etiological agents of anogenital and oropharyngeal carcinomas (12).

HPV16, a predominant high-risk variant, is intimately associated with oncogenesis, particularly cervical carcinogenesis (13, 14, 15). The viral genome comprises an approximately 8-kb circular double-stranded DNA encoding 8 canonical open reading frames (E1, E2, E4, E5, E6, E7, L1, and L2). These genes are functionally partitioned into early regulatory proteins (E1–E7), governing viral replication, transcription, and cellular transformation, and late structural proteins (L1, L2), constituting the viral capsid. The early proteins E1 and E2 orchestrate pivotal stages of the viral life cycle, including initiation of DNA replication, transcriptional regulation, and virion morphogenesis (16). E1 functions as a multifunctional helicase that recognizes the viral origin of replication (ori) and recruits host replication factors (17, 18). Conventional models posit E2 as a sequence-specific DNA-binding protein modulating transcriptional activation and suppression, while also stabilizing E1 and facilitating its nuclear localization (19, 20). Furthermore, E2 augments E1-mediated replication through direct protein–protein interactions (21, 22). Nevertheless, the autonomous enzymatic potential of E2 remains unexplored, and the precise stoichiometry and dynamics of E1–E2 complexes warrant further elucidation.

In this study, we developed a real-time duplex DNA unwinding assay leveraging Fluorescence Resonance Energy Transfer (FRET) to quantitatively characterize the helicase activity of HPV16 E1 and E2. We demonstrate that HPV16 E2 exhibits intrinsic ATP-dependent DNA unwinding activity, a function previously unassociated with this viral factor. The key residues at the ATP-binding site (Y303) and DNA-binding site (K299, Y303, and K306) of E2 are crucial for its ATPase and helicase activities. Mutations at these sites significantly impair both the ATPase and helicase activities of the E2 protein.

Podophyllotoxin (PPT) is a well-characterized antimitotic agent that inhibits microtubule polymerization by binding to the colchicine-binding site of tubulin, similar to colchicine and paclitaxel (23, 24, 25). Here, we identified an additional, previously unrecognized activity of PPT as direct inhibition of E2 helicase activity, with half-maximal inhibitory concentration (IC_50_) values in the nanomolar range. Crucially, E2 markedly attenuates E1-mediated unwinding kinetics in an interaction-dependent manner. Disruption of E2–E1 binding—achieved through interface mutations—abolished this transregulatory effect. Our work provides the first evidence of catalytic functionality in HPV16 E2, unveils a novel regulatory mechanism governing E1–E2 cooperativity, and identifies a pharmacologically actionable site for antiviral development. These insights advance fundamental understanding of HPV replication mechanics and offer new avenues for therapeutic intervention targeting viral helicase complexes.

## Results

### HPV16 E2 protein exhibits ATPase and helicase activities

**Figure 1:**
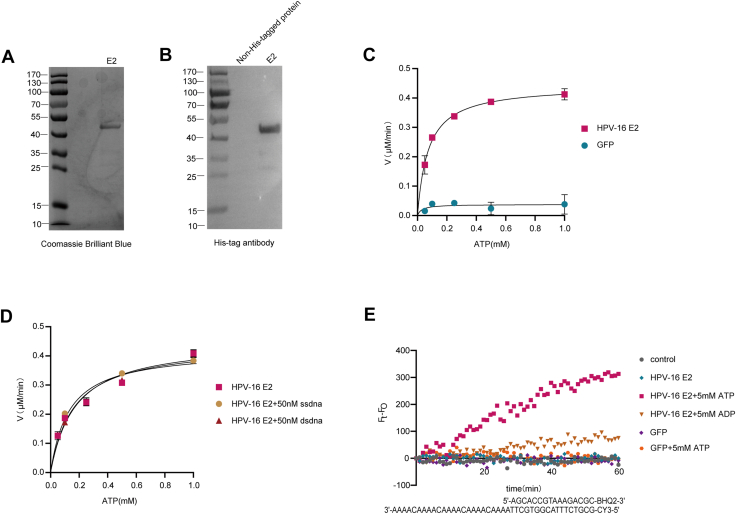
**Purification and enzymatic activity assays of HPV16 E2 protein.***A*, SDS-PAGE analysis of purified E2 protein. Proteins were visualized by Coomassie Brilliant Blue staining, with an empty lane containing no protein serving as a negative control. *B*, Western blot analysis of purified E2 protein using an anti-His antibody. A non-His-tagged protein was used as a negative control. *C*, ATPase activity of purified E2 protein. ATP hydrolysis assays were performed with 2.5 μM E2 protein or GFP protein (as a negative control) in the presence of the indicated ATP concentrations at 37 °C for 60 min. Data were fitted to the Michaelis-Menten equation and presented as a double-reciprocal plot. Error bars represent the standard error of replicate measurements. *D*, ATPase activity of HPV16 E2 protein in the absence or presence of ssDNA or dsDNA. Reactions contained 50 nM ssDNA (5′-AGCACCGTAAAGACGC-3′) or dsDNA (5′-AGCACCGTTAAAGACGC-3′ annealed to 3′-AAAACAAAAACAAAAACAAAAATTCGTGGGCATTTCTGCG-5′). Error bars represent the SEM from duplicate measurements. *E*, real-time unwinding kinetics of 0.4 μM E2 protein on a fluorescently labeled 16-bp dsDNA. Reactions were performed with 0.4 μM E2 protein in the presence or absence of 5 mM ATP or ADP, as indicated. Controls included GFP protein (0.4 μM) with or without 5 mM ATP, and buffer without protein. Fluorescence signals were measured every minute. Unwinding extent was defined as F_t_ − F_0_, where F_t_ is the fluorescence at time t and F_0_ is the initial fluorescence. The sequence information of the dsDNA is shown at the bottom of the corresponding assay plot.

The HPV E2 protein has long been considered a regulatory protein, and its intrinsic enzymatic activities remain to be fully characterized. To investigate the ATPase and helicase activities of HPV16 E2 protein, the full-length E2 protein (E2, residues 1–365) was expressed and purified from *Escherichia coli*. Coomassie Brilliant Blue staining and Western blotting confirmed that the protein exhibited the expected molecular weight, consistent with theoretical calculations (Fig. 1, *A* and *B*). As shown in Figure 1*A*, a distinct protein band corresponding to E2 was observed at the predicted molecular weight, while no protein bands were detected in the blank lane serving as the negative control. Western blot analysis further validated the specificity of the purified protein, revealing a single immunoreactive band at the expected molecular weight (Fig. 1*B*), whereas no significant signal was detected for the non-His-tagged protein used as a negative control. Helicases typically unwind nucleic acids using energy derived from ATP hydrolysis. We measured the ATPase activity of the E2 protein using a malachite green-based colorimetric assay, which detects free inorganic phosphate released during ATP hydrolysis. The data were fitted to the Michaelis-Menten equation by nonlinear regression (Fig. 1*C*). The results demonstrated that the purified full-length HPV16 E2 protein possesses ATP hydrolysis activity (Vmax = 0.52 μmol/L/min, Km = 0.11 mmol/L), whereas no significant ATPase activity was detected for the negative control GFP protein under identical conditions. Adding ssDNA or dsDNA substrate (50 nM) into the ATP hydrolysis reaction did not apparently affect E2 ATPase activity (Fig. 1*D*). Thus, HPV16 E2 has intrinsic ATPase activity without DNA binding. Next, the degree of DNA strand separation by HPV16 E2 protein was monitored in real time by FRET technology (Fig. 1*E*). Complementary nucleic acid strands were labeled with the fluorophore Cy3 or the quencher BHQ2. When the complementary strands annealed into a duplex, the fluorescence emitted by Cy3 was largely absorbed by BHQ2, resulting in a low Cy3 fluorescence signal. As the helicase unwound the duplex, the quenching effect of BHQ2 on Cy3 decreased or disappeared, leading to a corresponding increase in Cy3 fluorescence. As shown in Figure 1*E* a significant time-dependent increase in fluorescence was observed only in the reaction containing HPV16 E2 supplemented with ATP, indicating that the unwinding activity of E2 is strictly dependent on ATP hydrolysis. In contrast, only a weak increase in fluorescence was observed in the presence of E2 incubated with ADP, while no appreciable fluorescence enhancement was detected in the absence of E2, in reactions containing E2 without ATP, or in the GFP control group (regardless of ATP supplementation).

### Identification of key amino acid residues for HPV16 E2 protein enzymatic activity

**Figure 2:**
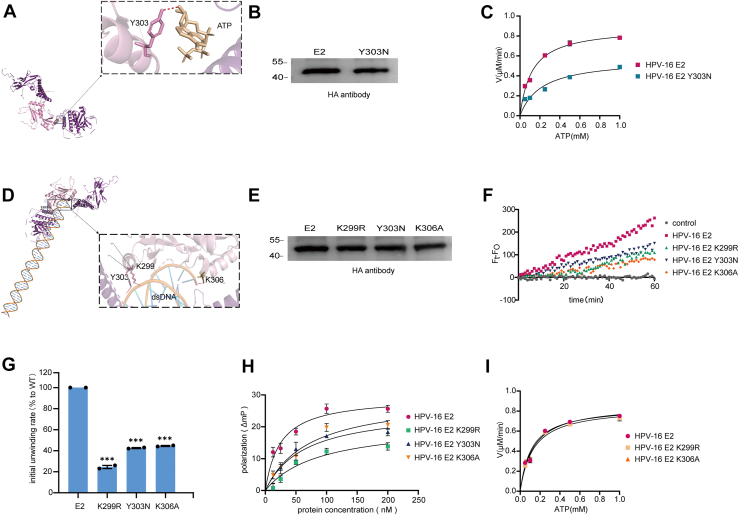
**Identification of key amino acid residues for HPV16 E2 protein enzymatic activity.***A*, molecular docking of the E2 dimer with ATP was performed using AutoDock, showing hydrogen bond formation between Y303 of one subunit and ATP. ATP is shown in *dark yellow*, Y303 in *pink*, and hydrogen bonds in *red*. *B*, Western blot analysis of purified wild-type E2 and mutant Y303N proteins using an anti-HA antibody. *C*, Comparison of ATPase activity between 2.5 μM wild-type E2 and mutant E2 Y303N. *D*, HDOCK-based docking of dimeric E2 protein with DNA, revealing hydrogen bonds between K299, Y303, K306, and DNA. K299 and Y303 are shown in *pink*, while K306 from the other monomer is shown in *dark brown*. DNA is represented in *dark yellow*, and hydrogen bonds are indicated in *red*. *E*, Western blot analysis of purified E2 protein and mutants K299R, Y303N, and K306A using an anti-HA antibody. *F*, real-time unwinding kinetics of 0.4 μM wild-type E2 and mutants K299R, Y303N, and K306A on fluorescently labeled 16-bp dsDNA. Buffer without protein served as a control.The no-protein control contained all components identical to the experimental reactions, including the same concentrations of substrate DNA and competitor DNA. Fluorescence signals were measured every 1 min. Unwinding extent was defined as F_t_ to F_0_, where F_t_ is the sample fluorescence at a given time and F_0_ is the initial fluorescence. *G*, quantification of the initial dsDNA unwinding rate of WT or mutated E2 in (*F*). The initial unwinding rate is calculated by a linear fit of the first 5 min of the reaction, and the initial unwinding rate of E2 is defined as 100%. Error bars represent the standard error of duplicate measurements (∗∗∗*p* < 0.001).Individual data points represent the duplicate measurements. *H*, fluorescence polarization assay to detect the binding of ssDNA and WT or mutated E2 (K299R, Y303N, and K306A). The assay was carried out with 50 nM ssDNA (3′-AAAACAAAAACAAAAACAAAAATTCGTGGGCATTTCTGCG-CY3-5′) in the presence of the indicated concentrations of enzyme. The data were fitted to a hyperbolic binding model, Kd_(E2)_ = 24 ± 8 nM, Kd_(K299R)_ = 88 ± 38 nM, Kd_(Y303N)_ = 64 ± 27 nM. Kd_(K306A)_ = 71 ± 27 nM. *I*, comparison of ATPase activity between 2.5 μM wild-type E2 and the K299R, K306A mutants.

Molecular docking was performed between the E2 dimer and ATP, followed by PyMOL visualization to identify interacting amino acid residues. Among these, residue Y303 in one subunit of the E2 dimer formed a hydrogen bond with ATP (Fig. 2*A*). This site was subsequently mutated, and Western blot analysis confirmed that the mutant protein exhibited the correct molecular weight, consistent with theoretical calculations (Fig. 2*B*). ATP hydrolysis assays revealed that the Y303N mutation significantly impaired the ATPase activity of E2 (Fig. 2*C*), indicating that Y303 is a critical residue for the ATPase function of E2. The E2 protein typically functions as a dimer when binding to DNA. Molecular docking of the dimeric E2 protein with DNA, followed by PyMOL visualization, identified additional interacting residues—K299, Y303, and K306—which formed hydrogen bonds with DNA (Fig. 2*D*). These sites were mutated, and Western blot analysis confirmed that the resulting mutant proteins had the expected molecular weights (Fig. 2*E*). Among different HPV genotypes, the catalytic residues K299 and Y303 are highly conserved, whereas K306 is not (Fig. S2). When the K299R, Y303N, and K306A mutants were subjected to dsDNA unwinding assays, they exhibited significantly reduced helicase activity and slower unwinding rates (Fig. 2, *F* and *G*). To determine whether this defect resulted from impaired DNA binding, we performed fluorescence polarization (FP) binding assays. The results showed that all three mutants exhibited markedly lower binding affinity to ssDNA compared to wild-type E2 (Fig. 2*H*). This indicate that mutations at these residues disrupt the DNA-binding ability of E2. We also performed Differential Scanning Fluorimetry (DSF) to verify whether these mutations affect protein stability. The results showed that the mutants exhibited melting profiles and Tm values similar to those of the wild-type protein, indicating that these mutations do not compromise protein stability (Fig. S7). We also measured the ATPase activity of the K299R and K306A mutants and found that the mutations at these sites did not affect their ability to hydrolyze ATP (Fig. 2*I*). Therefore, they reduce the unwinding activity by attenuating the interaction with DNA. We speculate that this residue, located near the helicase active region, may indirectly affect enzymatic function. These results demonstrate that these four amino acids are critical for the helicase activity of the E2 protein and further confirm that the enzymatic domain of E2 resides in its C-terminal region (residues 245–365).

### Podophyllotoxin (PPT) effectively inhibits E2 Protein helicase activity *In vitro*

**Figure 3:**
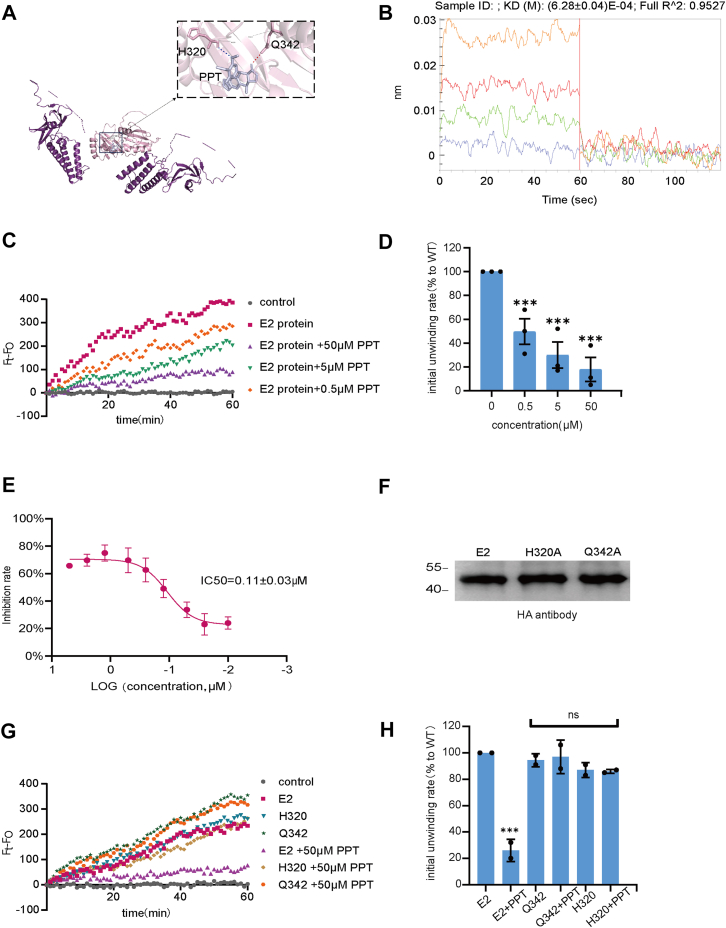
**Podophyllotoxin (PPT) effectively inhibits E2 protein helicase activity *in vitro*.***A*, molecular docking of the E2 protein with PPT using AutoDock. PPT is shown in *light purple*. Residues H320 and Q342 are shown in *pink*. The interaction between H320 and PPT (electrostatic repulsion) is indicated by a *blue dashed line*, while the hydrogen bond between Q342 and PPT is shown in *red*. *B*, bio-Layer Interferometry (BLI) analysis of the interaction between E2 protein and PPT. The curves from bottom to top represent working concentrations of PPT at 50, 100, 200, and 400 μM, respectively. Data were processed using double-referenced subtraction (sample and sensor reference) based on a 1:1 binding model. Kinetic parameters (k_on_ and k_off_) and the affinity constant (K_D_) were obtained using Octet BLI Analysis 12.2 software. *C*, real-time FRET-based detection of the inhibitory effect of PPT (0.5 μM, 5 μM, 50 μM) on the unwinding activity of 0.4 μM E2 protein. Buffer without protein served as a control. The no-protein control contained all components identical to the experimental reactions, including the same concentrations of substrate DNA and competitor DNA. *D*, quantification of the initial DNA unwinding rate of E2 in the presence of increasing concentrations of PPT (0.5, 5, and 50 μM) in (*C*). The initial unwinding rate is calculated by a linear fit of the first 5 min of the reaction, and the initial unwinding rate of E2 protein is defined as 100% (∗∗∗*p* < 0.001). Individual data points represent the triplicate measurements. *E*, determination of the IC_50_ value for PPT. Using the FRET assay, the reaction mixture was added to a white 96-well plate and covered with foil. After 1 h of incubation, fluorescence was measured using a microplate reader with excitation at 550 nm, emission at 620 nm, and a cutoff at 610 nm. The assay was performed with 0.4 μM E2 protein and PPT concentrations ranging from 0.1 mM to 0.00025 mM (0.1, 0.05, 0.025, 0.01, 0.005, 0.0025, 0.001, 0.0005, and 0.00025 mM). The experiment was repeated three times, and the IC_50_ was calculated using GraphPad Prism eight software. The graph displays the mean ± standard deviation of three independent experiments. *F*, Western blot analysis of purified wild-type E2 and mutant H320A, Q342A proteins using an anti-HA antibody. *G*, helicase assays measuring dsDNA unwinding by wild-type E2 or mutants H320A and Q342A in the presence or absence of PPT. Reactions contained 0.4 μM wild-type or mutant E2 proteins and 50 μM PPT, with 16-bp dsDNA as substrate. Buffer without protein served as a control. The no-protein control contained all components identical to the experimental reactions, including the same concentrations of substrate DNA and competitor DNA. *H*, quantification of the initial DNA unwinding rate of WT E2, Q320A, and H342A in the absence or presence of 50 μM PPT in (*G*). The initial unwinding rate is calculated by a linear fit of the first 5 min of the reaction, and the initial unwinding rate of E2 is defined as 100%. (∗∗∗*p* < 0.001); (ns, *p* > 0.05). Individual data points represent the duplicate measurements.

Podophyllotoxin (PPT) is a natural product currently used to treat external warts caused by HPV-6 and HPV-11 (26, 27, 28, 29, 30). It is well-established as an antimitotic agent that binds to microtubules, similar to colchicine and paclitaxel (23, 24, 25). However, whether PPT can directly interact with the HPV E2 helicase and inhibit its activity *in vitro* remains unknown. Molecular docking between the E2 dimer and podophyllotoxin (PPT) suggested their potential interaction. Residue Q342 of the E2 protein formed a hydrogen bond with PPT, while residue H320 exhibited electrostatic repulsion with the ligand (Fig. 3*A*). Bio-Layer Interferometry (BLI) was used to further validate this interaction, confirming that they bind to each other with an affinity (K_D_) of (6.28 ± 0.04) × 10^−4^ M (Fig. 3*B*). To investigate whether PPT inhibits the helicase activity of HPV16 E2 *in vitro*, a FRET-based assay was employed (Fig. 3, *C* and *D*). The inhibitory effect of PPT on E2 helicase activity was characterized by measuring the extent of dsDNA unwinding by E2 at increasing concentrations of PPT. The half-maximal inhibitory concentration (IC_50_) was determined to be IC_50_ = 0.11 ± 0.03 μM (n = 3) (Fig. 3*E*), indicating that PPT effectively inhibits the helicase activity of E2 *in vitro*. The conserved residues H320 and Q342 were mutated to alanine (Fig. S2),and the resulting mutant proteins were verified by Western blotting, which confirmed that their molecular weights matched the theoretical values (Fig. 3*F*). When these two residues were mutated, the inhibitory effect of PPT on helicase activity was abolished (Fig. 3, *G* and *H*), suggesting that H320 and Q342 are critical for the interaction between E2 and PPT. In conclusion, PPT inhibits the enzymatic activity of E2 by directly binding to the protein, establishing it as a small-molecule inhibitor of the E2 helicase.

### E2 Protein exhibits weaker enzymatic activity than E1 and inhibits E1 helicase activity

**Figure 4:**
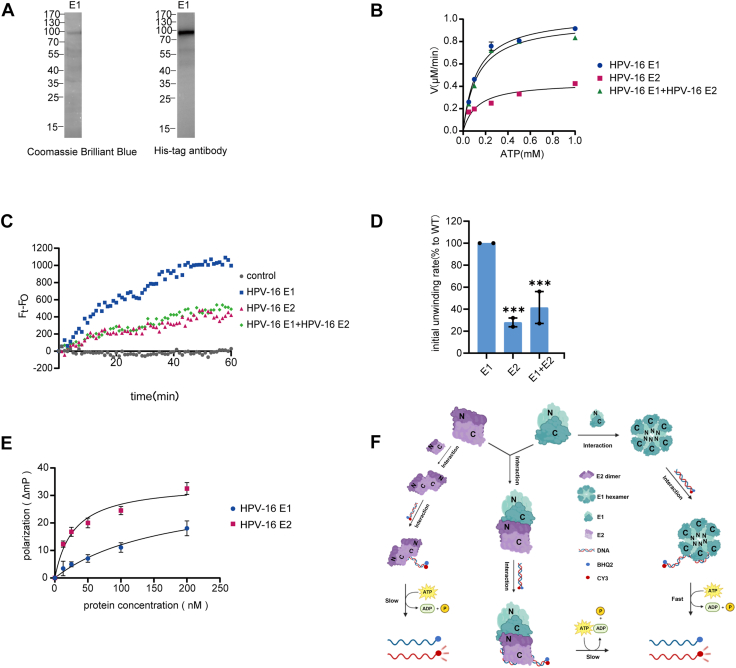
**Comparison of enzymatic activities between E2 and E1 proteins and examination of the effect of E2 on E1 activity.***A*, SDS-PAGE analysis of purified E1 protein stained with Coomassie *blue*. Western blot analysis of purified E1 protein using an anti-His antibody. *B*, ATPase activity of E2 protein alone and E1 protein with or without E2. ATP hydrolysis assays were performed at indicated ATP concentrations. Error bars represent the standard error of replicate measurements. *C*, Real-time unwinding kinetics of E1 alone, E2 alone, and E1 in the presence of E2 (each 0.4 μM) on fluorescently labeled 16-bp dsDNA. Buffer without protein served as a control. *D*, quantification of the initial DNA unwinding rate of HPV16 E1, E2, and E1 + E2 in (*C*). The initial unwinding rate is calculated by a linear fit of the first 5 min of the reaction, and the initial unwinding rate of E1 is defined as 100%. (∗∗∗*p* < 0.001). Individual data points represent the duplicate measurements. *E*, fluorescence polarization assay to detect the binding of ssDNA and WT E1 or E2. The assay was carried out with 50 nM ssDNA (3′-AAAACAAAAACAAAAACAAAAATTCGTGGGCATTTCTGCG-CY3-5′) in the presence of the indicated concentrations of enzyme. The data were fitted to a hyperbola, Kd_(E1)_ = 172 ± 88 nM, Kd_(E2)_ = 28 ± 10 nM. *F*, schematic model of E2-mediated regulation of E1 enzymatic activity. The model of E1 is presented as a hexamer. The N-terminal domain of E1 (residues 1–143) is shown in *light green*, while its C-terminal domain is in *dark green*. The N-terminal domain of E2 (residues 1–245) is depicted in *dark purple*, and its C-terminal domain (residues 245–365) in *light purple*. “Slow” indicates slow unwinding of dsDNA, while “Fast” indicates rapid unwinding.

Purified HPV16 E1 protein was confirmed by Coomassie blue staining and Western blot analysis to have the correct molecular weight, consistent with theoretical calculations (Fig. 4*A*). As previously established, HPV16 E1 protein assembles into a hexamer to exert its ATP-dependent helicase function (31, 32, 33, 34, 35, 36, 37). We compared the ATPase activities of E1 and E2 proteins using a malachite green-based colorimetric assay that detects free inorganic phosphate released during ATP hydrolysis. Data were fitted to the Michaelis-Menten equation by nonlinear regression (Fig. 4*B*), revealing significantly weaker ATPase activity in E2 compared to E1 (E2: Vmax = 0.44 μmol/L/min, Km = 0.12 mmol/L; E1: Vmax = 1.03 μmol/L/min, Km = 0.12 mmol/L). FRET-based assays comparing helicase activities showed that E2 unwound dsDNA at a markedly slower rate than E1, confirming its weaker helicase activity (Fig. 4, *C* and *D*). To determine whether the observed differences in helicase activity between E1 and E2 could be attributed to their intrinsic DNA-binding affinities, we performed fluorescence polarization (FP) binding assays using a CY3-labeled ssDNA probe. As shown in Figure 4*E*, E2 exhibited a substantially higher affinity for ssDNA. The result indicates that E2 possesses a significantly stronger intrinsic capacity to engage the DNA substrate compared to E1, despite its weaker helicase activity. Evidence indicates that the E2 protein inhibits the formation of the E1 hexamer through their direct interaction (37). We investigated whether E2 affects E1 activity. As shown in Figure 4*B*, the ATPase activity of E1+E2 group is similar to E1 alone. Because E2 itself also possesses ATPase activity, the results suggest that E2 may inhibit the ATPase activity of E1, which is consistent with previous reports (38). Additionally, E2 was found to inhibit E1 helicase activity (Fig. 4, *C* and *D*). We hypothesize that the N-terminal domain of the E2 protein (residues 1–245) binds to the C-terminal region of the E1 helicase (residues 143–645), thereby obstructing both its hexamer formation and its ability to bind the dsDNA substrate. Consequently, the E2 protein assumes the helicase function, binding to dsDNA and unwinding the double strand. The unwinding rate under these conditions is similar to the unwinding rate mediated by the E2 protein alone. When the E1 or E2 protein unwinds dsDNA independently, the unwinding rate of E1 is significantly faster than that of E2 (Fig. 4*F*).

### E2 inhibits E1 helicase activity through protein–protein interaction

**Figure 5:**
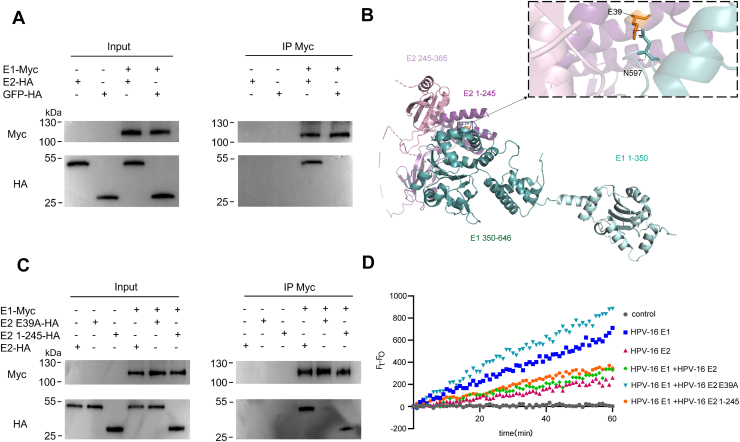
**E2 inhibits E1 helicase activity through protein-protein interaction.***A*, interaction between E2 and E1 proteins *in vitro*. After purification of E2-HA, E1-Myc, and the negative control GFP-HA in *E. coli* BL21, co-immunoprecipitation was performed, followed by Western blotting using anti-Myc or anti-HA antibodies. *B*, HDOCK-based prediction of key interaction sites between E1 and E2 proteins. The N-terminal domain of E2 (residues 1–245) is shown in *purple*, while its C-terminal domain (245–365) is in *pink*. The N-terminal domain of E1 (1–350) is depicted in *light green*, and its C-terminal domain (350–646) in *dark green*. In the figure, E39 is colored in *brown*, N597 is shown in *dark green*, and hydrogen bonds are depicted in *red*. *C*, Co-IP assay examining the interaction of E1 with wild-type (WT), mutant (E39A), and truncated E2 proteins (1–245), analyzed by Western blotting using anti-Myc or anti-HA antibodies. *D*, helicase assays measuring dsDNA unwinding by WT E2, E39A mutant, or truncated E2 (1–245) in the presence or absence of E1. Reactions contained 0.4 μM E2 (mutant or truncation) and E1, with 16-bp dsDNA as substrate.

To further validate whether E2 inhibits E1 helicase activity through direct interaction, we first confirmed the physical interaction between wild-type E2 and E1 proteins. Co-immunoprecipitation assays demonstrated that E1 specifically interacted with E2, but not with the negative control GFP, confirming the specificity of the E1-E2 interaction *in vitro*. (Fig. 5*A*). Molecular docking analysis revealed that the N-terminal domain of E2 (residues 1–245) binds to the C-terminal region of E1 (residues 143–645), with a specific hydrogen bond formed between E39 of E2 and N597 of E1 (Fig. 5*B*), confirming our previous hypothesis (Fig. 4*F*). Previous studies have established that the E39 residue of the HPV 11 E2 protein is essential for its replication function (39), and the structural basis for this lies in its direct participation in the interaction between the E2 activation domain and the E1 helicase (37). To disrupt the E2-E1 protein interaction, the conserved residue E39 was mutated to alanine (Fig. S2), and an N-terminal truncation mutant of E2 (residues 1–245) was designed to further validate the E1-binding domain. Western blot analysis confirmed proper expression of both E39A and N-terminal truncation proteins (Fig. S5*E*). Subsequent co-immunoprecipitation experiments showed that while the N-terminal truncation-maintained interaction with E1, the E39A mutation completely abolished this binding (Fig. 5*C*), demonstrating that E2 interacts with E1 through its N-terminal domain (1–245) and that E39 is a critical residue for this interaction. Using FRET-based real-time helicase assays (Fig. 5*D*), we observed that when the E39A mutation disrupted E2-E1 interaction, the unwinding activities of both proteins became additive. In contrast, the N-terminal truncation (1–245) and wild-type E2 maintained inhibition of E1 helicase activity, with unwinding kinetics similar to wild-type E2 (Fig. 5*D*). These results conclusively demonstrate that E2 inhibits E1 helicase activity through direct protein-protein interaction.

## Discussion

This study provides evidence through systematic biochemical and structural analyses that HPV 16 E2 protein possesses intrinsic ATPase and helicase activities. Mutagenesis studies identified residues critical for these activities (Y303 for ATPase; K299, Y303, and K306 for helicase), localizing the enzymatic domain to the C-terminal region (amino acids 245–365). These findings extend the conventional characterization of E2 as a transcriptional regulator to include potential direct involvement in viral DNA replication. While we have elucidated enzymatic features of E2 and its regulatory interplay with E1 helicase, several questions remain. It is currently unclear whether E2 maintains enzymatic activity in cellular or *in vivo* environments, and how mutations at critical residues affect HPV replication.

Of particular interest is the finding that podophyllotoxin (PPT), an existing anti-HPV therapeutic, inhibits E2 helicase activity through direct binding, with an IC_50_ value of 0.11 ± 0.04 μM. In addition to its well-established role as a microtubule polymerization inhibitor (23, 24, 25), our results suggest that PPT may directly target the E2 helicase. This observation raises the possibility of developing inhibitors that target this viral protein. Compared to existing E6/E7-targeting inhibitors (*e.g.*, ciclopirox, C646, REBACIN) (40, 41, 42), targeting E2 could potentially block viral replication at an earlier stage. However, whether this newly identified mechanism contributes to the known anti-HPV effects of PPT remains to be determined. It is possible that PPT exerts multiple effects against HPV, targeting both host cell division and viral replication. Future studies using PPT analogs that lack antimitotic activity will be necessary to assess the contribution of E2 inhibition to the overall anti-HPV effects.

Physiological implications of the weak helicase activity of E2 and its regulatory role in replication initiation. The observation that E2 possesses intrinsic but weak helicase activity raises a question regarding its role in the viral life cycle. Based on these findings, we propose two possible functions for E2 during HPV replication initiation. First, prior to the recruitment of E1 and the assembly of an active double-hexameric helicase, the origin DNA may undergo initial localized melting. The weak helicase activity of E2 could function at this stage, potentially acting as an initiator that facilitates local melting or structural inspection of the origin region. Such activity might help ensure that the DNA conformation is suitable for efficient E1 loading and subsequent unwinding. The relatively weak activity could be advantageous in this context, as it may help prevent premature or nonspecific unwinding while potentially enhancing the fidelity of replication initiation.

Second, E2 may function as a temporal regulator of E1 activity. It possesses DNA-independent ATPase and weak helicase activities, and it hydrolyzes ATP continuously regardless of DNA binding, providing a low level of unwinding that may assist local melting at the origin or serve as a structural checkpoint. When E2 is recruited to the replication complex, its N-terminal domain (amino acids 1–245) binds to the helicase domain of E1, with E39 forming a critical hydrogen bond with N597. This physical interaction has dual effects. (1) steric hindrance that interferes with E1 hexamer assembly and reduces its binding to the DNA substrate; and (2) a decrease in E1 ATPase activity and a restriction of the overall unwinding rate to a low level comparable to that of E2 alone. This protein-interaction-dependent braking mechanism prevents premature or nonspecific unwinding by E1, ensuring that robust helicase activity is only achieved upon assembly of a complete replication complex. Disruption of the E2–E1 binding interface (*e.g.*, the E39A mutation) relieves this inhibition and restores E1 unwinding activity to an additive level. Thus, E2 acts as a bifunctional factor, a DNA-independent ATPase/helicase that can pre-initiate replication origins, and a protein-interaction-dependent brake that safeguards the correct temporal ordering of HPV DNA replication. Collectively, these findings support a temporal regulatory model in which E1 drives early genomic amplification, while increasing levels of E2 subsequently attenuate E1 activity to modulate replication frequency, potentially averting excessive replication that could otherwise trigger host defense responses.

## Experimental procedures

### Reagents

All reagents used in the experiments include: Anti-HA Tag Mouse Monoclonal Antibody (Selleck), Anti-MYC Tag Mouse Monoclonal Antibody (Selleck), Anti-His Tag Mouse Monoclonal Antibody (5C3) (Abbkine), ATP (CAS: 56-65-5, Solarbio), Podophyllotoxin (CAS: 518-28-5, Aladdin).

### Plasmid Construction and mutagenesis

HPV 16 E1/E2 sequences were amplified using gene-specific primers and inserted into designated vectors *via* restriction enzyme digestion and ligation. Site-directed mutagenesis primers were designed based on the wild-type template sequence, targeting upstream and downstream regions of the desired mutation sites. Primer parameters were optimized using online tools (*e.g.*, QuikChange Site-Directed Mutagenesis Manual) to ensure matching Tm values and correct placement of mismatched bases. Mutants and truncations of E2 were generated through site-directed mutagenesis. All constructs were verified by sequencing.

### Recombinant protein expression and purification

All recombinant proteins were expressed in *E. coli* BL21. Cells transformed with HPV 16-pET28a plasmids were cultured in Luria-Bertani medium containing kanamycin (100 μg/ml) at 37 °C until OD_600_ reached 0.8. Protein expression was induced with 0.4 mM isopropyl-β-D-thiogalactopyranoside (IPTG) at 16 °C for 24 h. Cells were harvested, resuspended in lysis buffer (20 mM Tris-HCl, 500 mM NaCl, 0.5% Triton X-100, 20 mg/L RNase A, 20 mg/L DNase I, 10% glycerol, 1 mM DTT, protease inhibitors, pH 8.0), and lysed using a high-pressure homogenizer at 4 °C (1300 Pa, seven cycles). The lysate was centrifuged at 18,000 rpm for 45 min at 4 °C. His-tagged proteins were purified from the supernatant using Ni-NTA agarose beads with gentle inversion mixing for 4 h at 4 °C. Discard the supernatant and transfer the beads to a binding column. Add wash buffer (20 mmol/L Tris-HCl, 500 mmol/L NaCl, 0.4 mmol/L DTT, 30 mmol/L imidazole, pH 8.0), mix well, and incubate on ice for 3 min. Then, drain the liquid from the column. Repeat the above operation. Continue washing the beads with a higher concentration of imidazole solution (20 mmol/L Tris-HCl, 500 mmol/L NaCl, 0.4 mmol/L DTT, 60 mmol/L imidazole, pH 8.0) until the effluent no longer turns blue upon addition of Coomassie Brilliant Blue G250 staining solution. After washing, completely drain the liquid from the beads. Add elution buffer (20 mmol/L Tris-HCl, 500 mmol/L NaCl, 500 mmol/L imidazole, 1 mmol/L DTT, 10% glycerol, pH 8.0), mix well, and incubate on ice for 5 min. Collect the liquid from the column. The eluate was dialyzed twice against buffer (100 mM NaCl, 20 mM Tris-HCl pH 8.0, 10% glycerol, 1 mM DTT) for 12 and 4 h, respectively. Proteins were concentrated to approximately 0.8 mg/ml using 30/50 kDa cutoff concentrators (Solarbio), flash-frozen in liquid nitrogen, and stored at −80 °C.

### Protein structure prediction and molecular docking

HPV16 E1/E2 protein sequences were retrieved from http://www.ncbi.nlm.nih.gov/. Three-dimensional structures were predicted using https://alphafold3.org/. Protein interactions with small molecules, nucleic acids, or other proteins were predicted *via* http://hdock.phys.hust.edu.cn/or AutoDock software. The resulting PDB files were visualized using PyMOL.

### ATPase activity assay

ATPase activity was measured using the QuantiChrom ATPase/GTPase Assay Kit (BioAssay Systems) to quantify inorganic phosphate released from ATP hydrolysis. Phosphate standards were prepared according to the manufacturer's instructions. Reactions were performed in 96-well plates with 40 μl reaction volume per well. Wild-type or mutant HPV16E proteins (0.4 μM) were incubated with varying ATP concentrations in buffer (20 mM Tris, 40 mM NaCl, 4 Mg(AcO)_2_, 0.5 mM EDTA, pH 7.5) at 37 °C for 1 h. Dialysis buffer without protein served as the control. Reactions were terminated by adding 200 μl malachite green reagent, incubated for 30 min at room temperature, and absorbance was measured at 620 nm using a microplate reader. Reaction velocities and ATP concentrations fit the Michaelis-Menten equation: $V=\frac{Vmax\left[S\right]}{Km+\left[S\right]}$

### Helicase activity assay

Helicase activity was assessed using a FRET-based dsDNA unwinding assay. Complementary DNA oligonucleotides labeled with Cy3 or BHQ2 were synthesized and dissolved in annealing buffer (20 mM Tris-NaCl, 2 mM MgCl_2_, 0.2 mM DTT, 100 mM KCl, pH 7.5) to 10 μM. dsDNA was prepared by mixing equimolar complementary strands to a final concentration of 500 nM, heating to 95 °C for 5 min, and slowly cooling to room temperature. Unwinding reactions contained 0.4 μM purified HPV16 E2 protein, 50 nM dsDNA substrate, 5 mM ATP, and 500 nM unlabeled competitor DNA(5′-GCGTCTTTACGGTGCT-3′) in reaction buffer (20 mM Tris-HCl pH 7.0, 10 mM NaCl, 0.1 mg/ml BSA, 5 mM MgCl_2_, 2 mM DTT) in a total volume of 100 μl. As a negative control, a mock reaction without protein (control) was prepared containing all the identical components, including the same concentrations of dsDNA substrate and unlabeled competitor DNA. Reactions were transferred to white 96-well plates, covered with foil, and fluorescence was measured every minute for 1 h using a microplate reader (excitation: 550 nm, emission: 620 nm, cutoff: 610 nm).

### Co-immunoprecipitation

E1 and E2 Proteins were mixed at a 1:1 M ratio in binding buffer (0.5% Triton X-100 in PBS) and incubated with rotation at 4 °C for 2 h. MYC antibody was added and incubated for another 2 h, followed by the addition of 20 μl Protein A/G beads and further rotation for 2 h. Beads were pelleted by centrifugation at 12,000 rpm for 2 min at 4 °C, washed three times with wash buffer (20 mM Tris–HCl, 300 mM NaCl, 0.1 mM EDTA, pH 8.0), resuspended in 1× protein loading buffer, boiled for 10 min, and centrifuged. The supernatant was collected for Western blot analysis.

### Bio-Layer Interferometry (BLI)

Bio-Layer Interferometry (BLI) was employed to quantitatively characterize the binding interaction between HPV16 E2 protein and PPT. This technique utilizes fiber optic biosensors to monitor changes in the optical thickness of the sensor layer resulting from biomolecular binding events (43, 44, 45, 46). In this system, analytes interacting with ligands immobilized on the sensor surface form a monolayer, leading to proportional shifts in the interference spectrum of reflected light. Analyses were performed on a ForteBio Octet RED96 system equipped with dedicated data acquisition and analysis software (Pall ForteBio Corp.). All steps were carried out at 25 °C with a total working volume of 200 μl for samples and buffers, loaded in the instrument-specific 96-well black plates. Prior to measurement, Ni-NTA sensors were pre-wet in protein storage buffer (100 mM NaCl, 20 mM Tris-HCl pH 8.0, 10% glycerol, and 1 mM DTT) for 10 min. The loading procedure consisted of three steps: (1) baseline (60 s), (2) loading (60 s), and (3) baseline (60 s). After loading, the Ni-NTA biosensors bound with E2 protein and control sensors were equilibrated in assay buffer before being transferred into wells containing different concentrations of podophyllotoxin for binding measurement.TBS-T (137 mM NaCl, 2.7 mM KCl, 25 mM Tris, 0.05% Tween-20) served as the assay buffer, with podophyllotoxin concentrations tested at 50, 100, 200, and 400 μM. The binding assay protocol comprised five steps: (1) baseline (300 s), (2) protein association (120 s), (3) baseline (60 s), (4) small molecule association (60 s), and (5) dissociation (60 s). Finally, kinetic parameters (kon and koff) and the affinity constant (K_D_) were obtained using a double-referencing subtraction method (sample and sensor reference) based on a 1:1 binding model, processed with the data analysis software (Octet BLI Analysis 12.2).

### IC_50_ determination of PPT as an E2 helicase inhibitor

PPT was serially diluted in DMSO to concentrations ranging from 0.1 mM to 0.0001 mM. E2 protein (0.4μM) was pre-incubated with DMSO or PPT for 20 min, followed by addition of reaction buffer containing 50 nM dsDNA substrate, 4 mM ATP, and 500 nM competitor DNA. Reactions were carried out in white 96-well plates (100 μl total volume) covered with foil. After 1 h, fluorescence was measured (excitation: 550 nm, emission: 620 nm, cutoff: 610 nm). Experiments were performed in triplicate. Inhibition rate was calculated as:

$$Inhibition\%=\left(1-\frac{\left(Protein+PPT\right)-Control}{\left(Protein\right)-Control}\right)*100\%$$

Data were fitted using GraphPad Prism 8 with the “log(inhibitor) vs. response – Variable slope (four parameters)” model.

### Fluorescence polarization (FP)

Fluorescence polarization assay was performed by using 50 nM Cy3-labeled DNA (3′-AAAACAAAAACAAAAACAAAAATTCGTGGGCATTTCTGCG-CY3-5′) and the indicated concentrations of protein (0–200 nM) in the following buffer: 20 mM Tris–HCl pH 7.0, 10 mM NaCl, 0.1 mg/ml BSA, 5 mM MgCl_2_, 1 mM DTT. Upon protein binding, the increase in molecular rotational correlation time leads to an increase in the measured fluorescence polarization signal. Reaction mixtures were incubated at room temperature for 60 min to reach binding equilibrium. Fluorescence readings were taken using a plate reader (Flexstation3, Molecular Devices) with the following settings: E_x_ 530 nm, E_m_ 570 nm, cutoff 570 nm. All experiments were performed with three technical replicates. The equation for fitting FP data was $Y=\frac{\text{Bmax}\times X\;}{\text{Kd}+X}$, where Y is the fluorescence polarization signal, X is the protein concentration, Bmax is the maximum specific binding signal, and Kd is the equilibrium dissociation constant. The data were fitted to a one-site specific binding model using nonlinear regression in GraphPad Prism eight software to obtain the Kd values and standard errors.

## Data availability

This study includes no data deposited in external repositories.

## Supporting information

This article contains supporting information.

## Conflict of interest

The authors declare that they have no conflicts of interest with the contents of this article.
