# Supplementary material for: HPV16 E2 protein possesses intrinsic helicase activity and sterically hinders E1 function through direct interaction
Source: J Biol Chem. 2026 May 20;302(7):113143. doi: 10.1016/j.jbc.2026.113143 (PMC13284460; doi:10.1016/j.jbc.2026.113143)
Supplement: Figures S1–S8 [file mmc1.docx]

**Supplementary Data**

S1


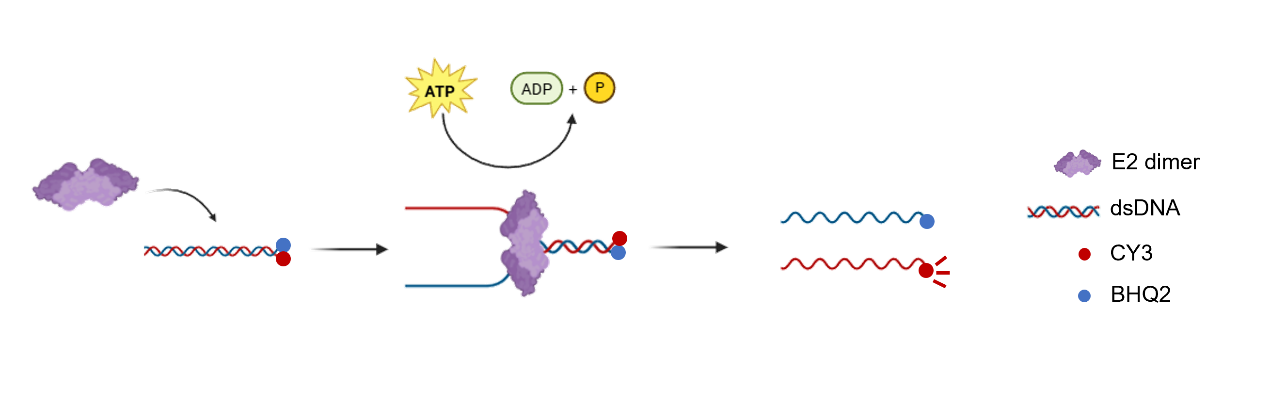


Figure S1. Schematic diagram of the fluorescence resonance energy transfer (FRET)-based DNA unwinding assay. Two complementary nucleic acid strands are labeled with a Cyanine 3 fluorophore (Cy3)and a Black Hole Quencher 2 (BHQ2), respectively. When the complementary strands are annealed to form a duplex, the fluorescence emitted by Cy3 is quenched by BHQ2. As the E2 protein unwinds the duplex with energy supplied by ATP hydrolysis, the quenching effect between Cy3 and BHQ2 is eliminated, leading to an increase in Cy3 fluorescence intensity.

S2


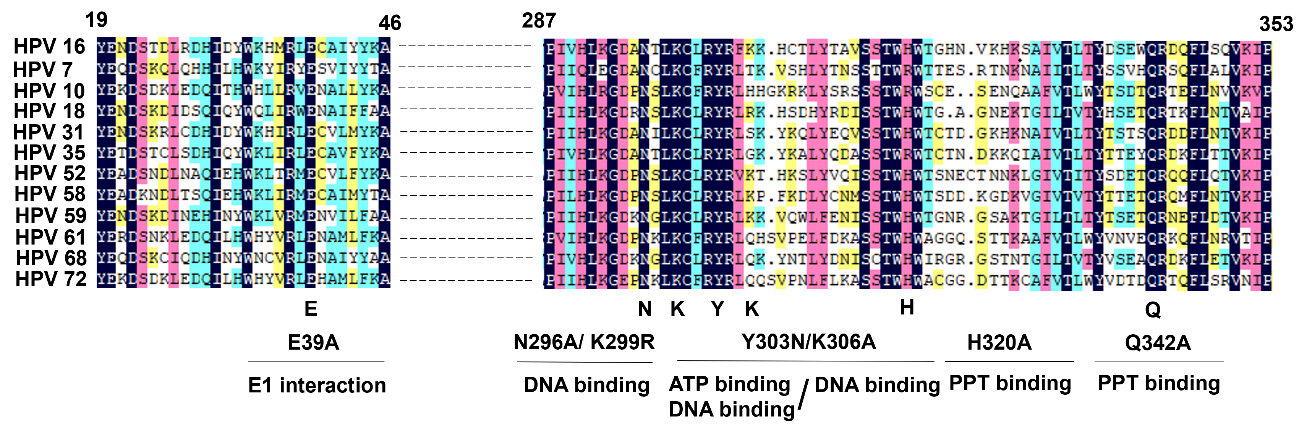


Figure S2. Amino acid sequence alignment of E2 proteins from multiple HPV types. The low-risk HPV types included in this alignment are: HPV7 (GenBank: QEE83811.1), HPV10 (NCBI Reference Sequence: NP_041744.1), HPV61 (GenBank: WAB54486.1), and HPV72 (GenBank: CAA63876.1). The high-risk HPV types included are: HPV16 (GenBank: AGO86724.1), HPV18 (GenBank: AGO86741.1), HPV31 (GenBank: AGO86736.1), HPV35 (GenBank: WAB53994.1), HPV52 (GenBank: ACX32360.1), HPV58 (GenBank: ACX32372.1), HPV59 (GenBank: ACL12337.1), and HPV68 (GenBank: ACX32380.1). Residues E39, N296, K299, Y303, H320 and Q342 are highly conserved across all HPV types, while residue K306 are not conserved. The mutated residues investigated in this study are indicated at the bottom of the sequences.

S3


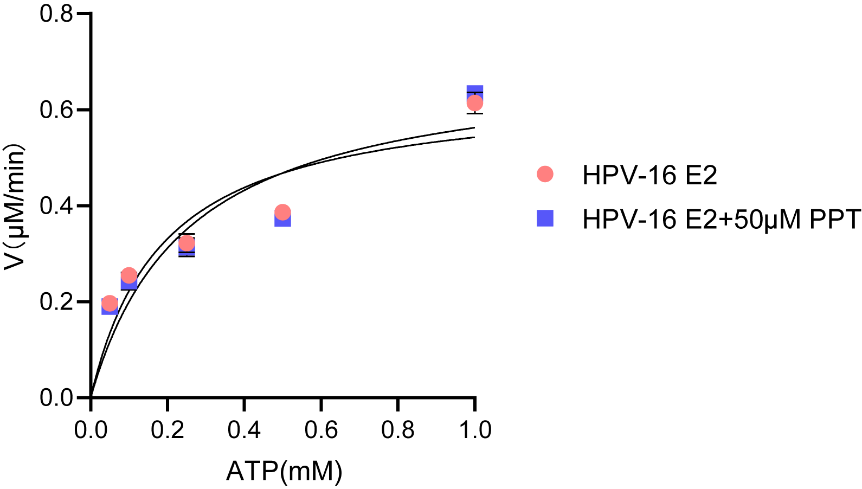


Figure S3. Detection of whether the ATPase activity of E2 is affected in the presence of PPT. It was found that the extent of ATP hydrolysis by E2 protein in the presence of PPT was consistent with that of E2 protein alone, indicating that PPT does not affect the ATPase activity of E2 protein. The reaction system contained 50 μM PPT and 0.4 μM E2 protein.

S4

Figure S4. Real-time unwinding kinetics of 0.4 μM wild-type E2 protein and mutant variants K299R and N296A on a fluorescently labeled 16 bp dsDNA. When the K299R and N296A mutants were applied in the dsDNA unwinding assay, they exhibited significantly reduced helicase activity and slower unwinding rates. Thus, the conserved residue N296 is also essential for the helicase activity of the E2 protein.

S5


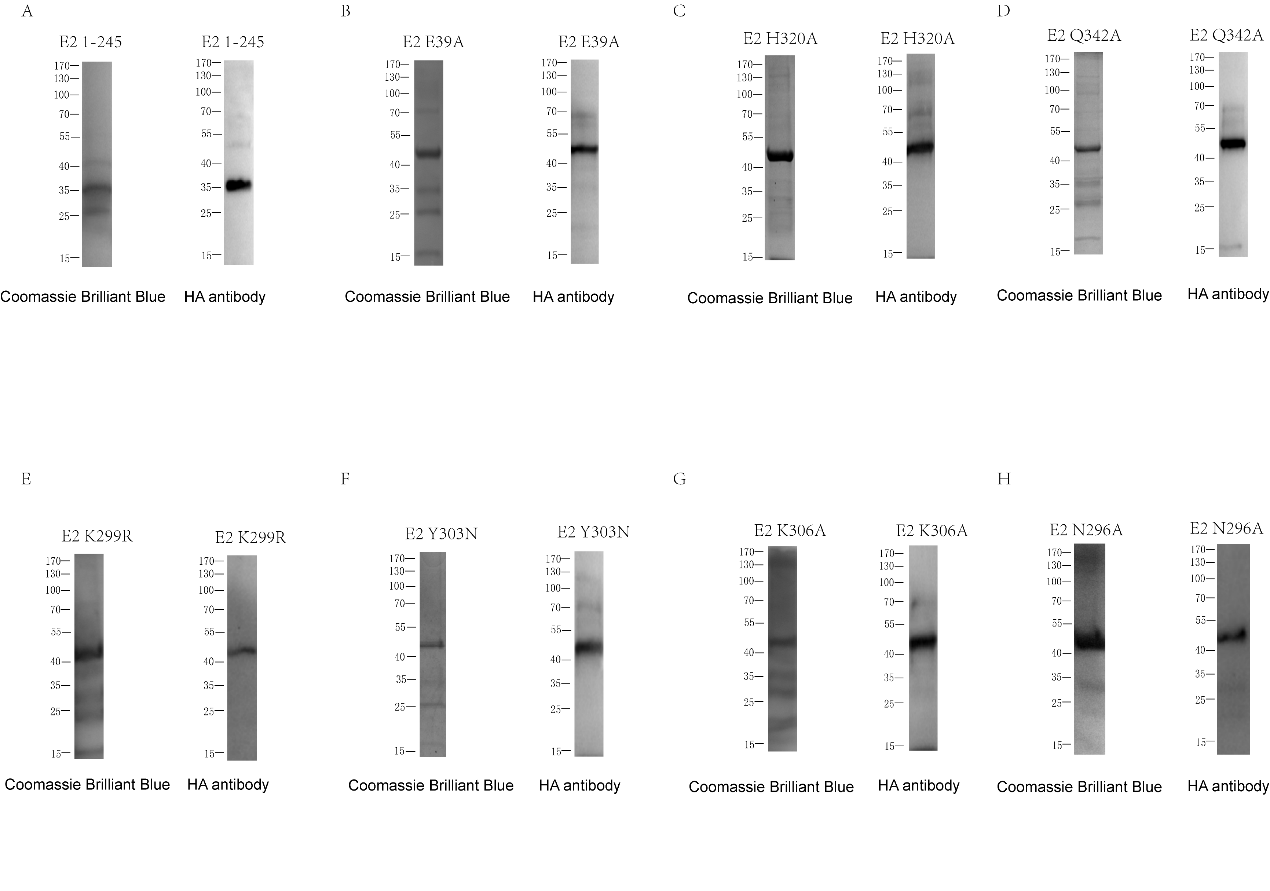


Figure S5. Purified E2 protein mutants and truncations. Western blot analysis shows that the molecular weights of the proteins are correct and consistent with theoretical calculations. (A) SDS-PAGE analysis of the purified E2 protein truncation (1-245), stained with Coomassie Brilliant Blue. Western blot analysis was performed using an anti-His antibody.(B) SDS-PAGE analysis of the purified E2 protein mutant E39A, stained with Coomassie Brilliant Blue. Western blot analysis was performed using an anti-His antibody.
(C) SDS-PAGE analysis of the purified E2 protein mutant H320A, stained with Coomassie Brilliant Blue. Western blot analysis was performed using an anti-His antibody. (D) SDS-PAGE analysis of the purified E2 protein mutant Q342A, stained with Coomassie Brilliant Blue. Western blot analysis was performed using an anti-His antibody.(E) SDS-PAGE analysis of the purified E2 protein mutant K299R, stained with Coomassie Brilliant Blue. Western blot analysis was performed using an anti-His antibody.(F) SDS-PAGE analysis of the purified E2 protein mutant Y303A, stained with Coomassie Brilliant Blue. Western blot analysis was performed using an anti-His antibody.(G) SDS-PAGE analysis of the purified E2 protein mutant N296A, stained with Coomassie Brilliant Blue. Western blot analysis was performed using an anti-His antibody.(H) SDS-PAGE analysis of the purified E2 protein mutant K306A, stained with Coomassie Brilliant Blue. Western blot analysis was performed using an anti-His antibody.

S6


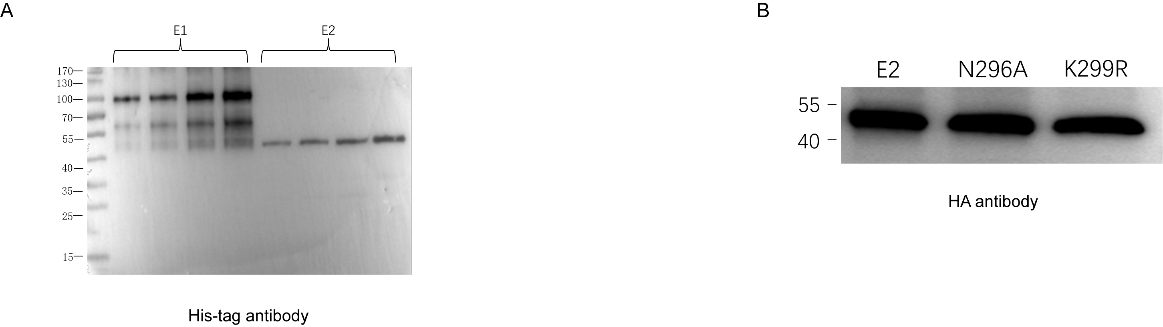


Figure S6. Western blot analysis of wild-type E1 protein and E2 mutants compared with wild-type E2 protein. (A) Western blot analysis of wild-type E1 and E2 proteins. Western blot analysis confirmed that the mutant protein exhibited the correct molecular weight, consistent with theoretical calculations. (B) Western blot analysis of E2 protein mutants N296A and K299R compared with wild-type E2 protein. Western blot analysis confirmed that the mutant protein exhibited the correct molecular weight, consistent with theoretical calculations.

S7


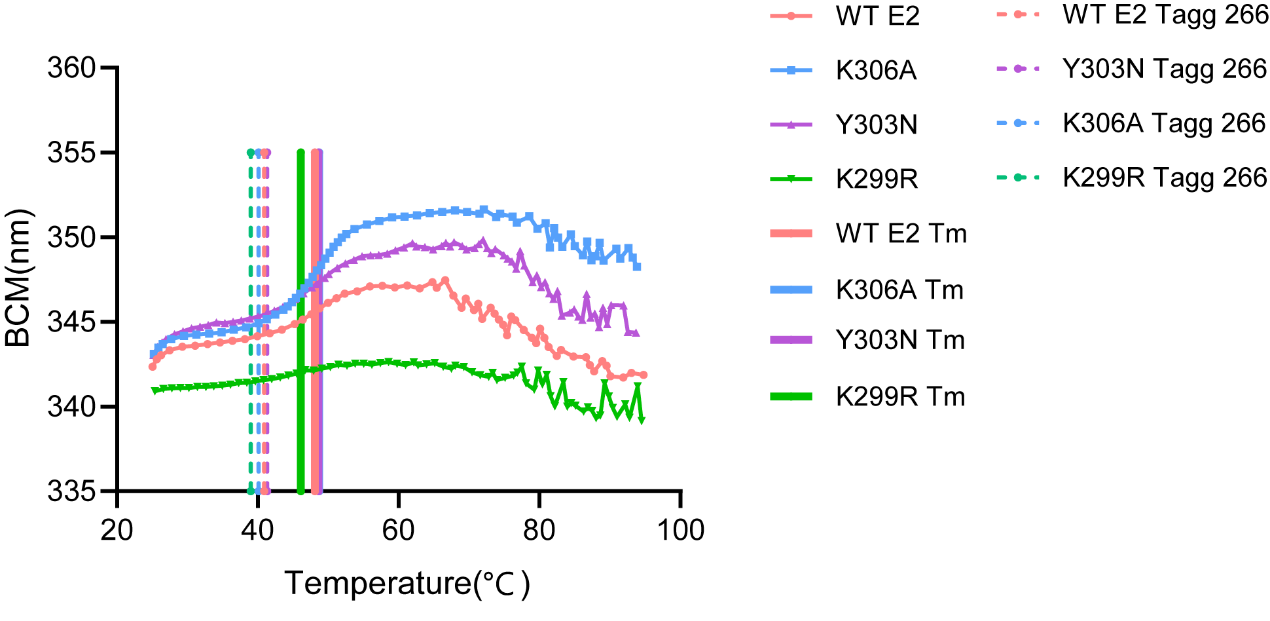


Figure S7. Thermal denaturation analysis of wild-type and mutant E2 proteins. The thermal stability of purified wild-type E2 and its mutants (K299R, Y303N, and K306A) was assessed by monitoring changes in fluorescence signal (BCM/nm) over a temperature gradient from 20°C to 100°C. The melting temperature (Tm) for each protein was determined from the inflection point of the denaturation curve. All three mutants exhibited melting profiles and Tm values comparable to those of wild-type E2, indicating that the point mutations did not disrupt the overall structural integrity or stability of the E2 protein.

S8


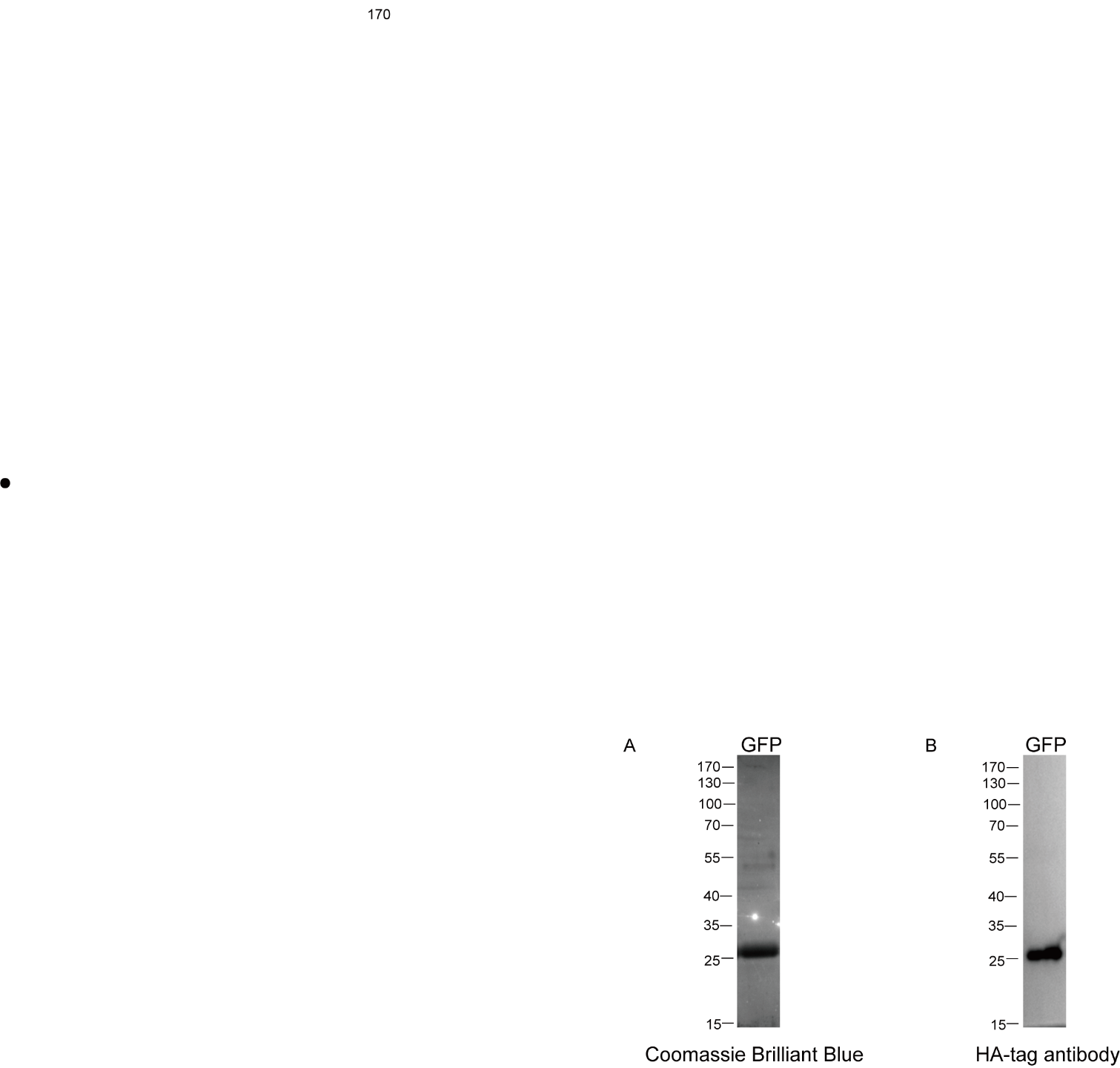


Figure S8. Purified GFP protein. Western blot analysis shows that the molecular weights of the proteins are correct and consistent with theoretical calculations. (A) SDS-PAGE analysis of the purified GFP protein, stained with Coomassie Brilliant Blue. (B)Western blot analysis was performed using an anti-HA antibody.
